# Taking aim with IAP antagonists at triple-negative breast cancer: a moving target no more?

**DOI:** 10.1038/s41419-020-2533-x

**Published:** 2020-05-11

**Authors:** Eric C. LaCasse

**Affiliations:** Apoptosis Research Centre, Children’s Hospital of Eastern Ontario Research Institute, Ottawa, ON K1H 8L1 Canada

**Keywords:** Apoptosis, Breast cancer

Triple-negative breast cancer (TN-BC) is a malignancy with the worst prognosis for all four corresponding stages compared with other receptor-positive BCs^1^. TN-BC account for 10–20% of all BCs, with BC affecting one in nine women over their lifespan. TN-BC is defined by immunohistochemistry as lacking the estrogen receptor (ER-negative), the progesterone receptor that is normally induced by functional estrogen receptor (PR-negative) and lacking the human epidermal growth factor 2 (Her2) receptor overexpression or amplification as well (Her2-negative). Hence, TN-BC is nonresponsive to targeted BC therapies such as small-molecule endocrine therapies typified by anti-estrogens (ER antagonists) or aromatase inhibitors (that block conversion of endogenous androgens to estrogens), and alternatively to biologics that target the Her2 plasma membrane receptor. These targeted therapies have become mainstays in BC treatment and adjuvant therapy resulting in improved survival for those receptor-positive BC patients. Traditional cytotoxic chemotherapy, consisting of taxanes and other agents, remains the only therapeutic option for metastatic TN-BC. The limited success with these drugs drives the need for new effective therapies for this “untargetable” BC.

## Not all TN-BCs are the same

**Fig. 1:**
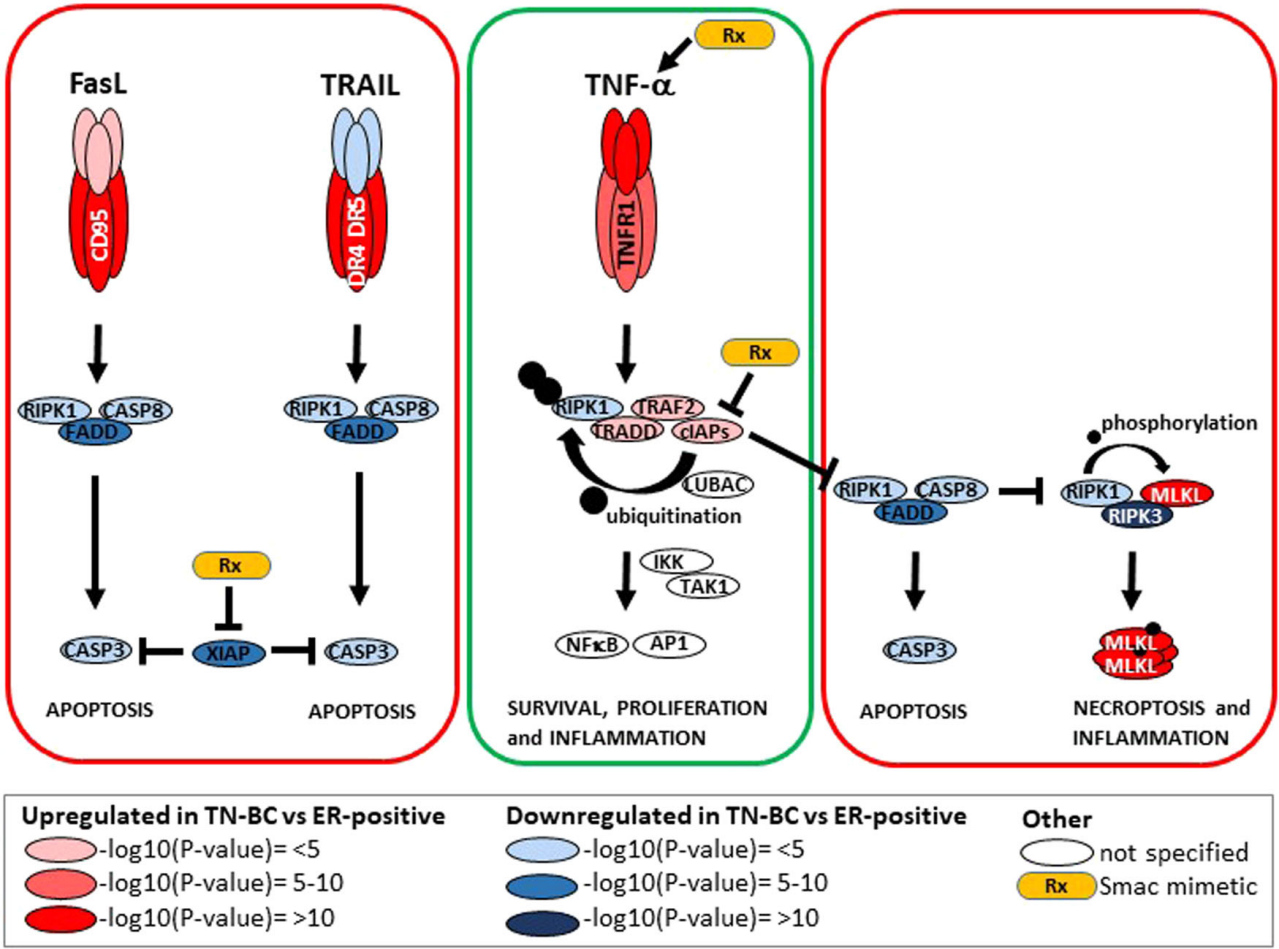
Differential expression of TNF superfamily pathway components underlies the killing of TN-BC by Smac mimetics compared with ER-positive BC. Schematic of death receptor signaling pathways for TNF-α, FasL, and TRAIL with ultimate cell fate outcomes shown (survival outcomes boxed in green, death outcomes boxed in red). Also indicated in three levels of red fill color (based on RNA −log10 *p* values) are components that are upregulated in the TCGA database for TN-BC compared with ER-positive BC. While three levels of blue fill color (based on RNA −log10 *p* values) are components that are downregulated in the TCGA database for TN-BC compared with ER-positive BC. Components in white fill are not indicated in the report^6^. The yellow drug capsule represents the small-molecule IAP antagonists known as Smac mimetics (SMs), which have multiple effects illustrated here. SMs induce the degradation of cIAP1 and cIAP2 thereby stopping TNF-α/TNFR1 triggered RIPK1 ubiquitination that blocks NF-κB and AP-1 activation and shunts RIPK1 into death complexes, referred to as the ripoptosome (RIPK1, FADD, and caspase-8) or the necrosome (RIPK1, RIPK3, and MLKL). SMs can also derepress XIAP’s ability to inhibit caspase-3, which sensitizes cells to FasL- or TRAIL-induced killing. SMs can also cause the induction of TNF-α through the activation of the alternative NF-κB pathway (not illustrated here). Activated caspase-8 is able to suppress necroptosis by cleavage of RIPK1 and RIPK3 proteins.

The pathological classification of TN-BC as an apparent single entity belies the fact of TN-BC’s heterogeneity that represents many different histological types of cancers, including basal-like, claudin-low, luminal A, luminal B, Her2-enriched, or metaplastic BC^2^. Specific genetic abnormalities are seen within certain TN-BC cohorts, such as those patients with the BRCA1/2 familial breast cancer gene mutations that are responsive to PARP inhibitors, which does support the concept of targeting TN-BCs with specific cancer agents^3^. The TN-BC cell line MDA-MB-231 is exquisitely sensitive to killing by investigational targeted agents, called Smac mimetics (SMs), which target several key members of the inhibitor-of-apoptosis (IAP) family possessing E3 ubiquitin ligase activity. The MDA-MD-231 cell line produces the cytokine tumor necrosis factor alpha (TNF-α) autonomously and this is required for cell killing by SMs. The SMs induce the ubiquitination and degradation of two fungible IAPs, cIAP1 and cIAP2, altering TNF-α signaling pathways in cancer cells away from survival and inflammatory MAPK and classical NF-κB and AP-1 signaling toward the induction of apoptotic (RIPK1 and caspase-8 dependent) or necroptotic cell death (RIPK1, RIPK3, and mixed-lineage kinase-like (MLKL) dependent) pathways (Fig. 1)^4^. The cIAPs ubiquitinate RIPK1 at the TNF receptor (TNFR1) upon TNF-α engagement and promote the formation of a signaling complex (involving the recruitment of another ubiquitin ligase, LUBAC, as well as several kinases that ultimately activate MAP kinases and the transcription factors NF-κB and AP-1). In addition, the cIAPs suppress the formation of RIPK1 death complexes that occurs when TNFR1 is engaged and RIPK1 is deubiquitinated. Some SMs, especially at higher doses, also target the potent and direct caspase inhibitor, the X-linked IAP (XIAP), and this drug binding competes with XIAP’s ability to suppress the death proteases, caspases-3, -7, and -9. The SM inhibition of XIAP is known to also sensitize to cell death induced by specific death ligands from the immune system, notably TRAIL and FasL/CD95L (e.g.^5^).

In the report by Lalaoui et al., the subject matter of this commentary, the authors test the activity of potent dimeric SMs, exemplified by birinapant, against a panel of TN-BC and ER-positive BC patient-derived xenograft cell lines and established cell lines for viability and tumor growth^6^. The TN-BCs were sensitive to SM killing in vitro, while the ER-positive BCs were resistant, and this translated to a reduction in tumor growth and an increase in mouse survival times for the SM-treated TN-BCs. The investigators undertook a survey and mechanistic evaluation of some of the key factors involved in the IAP-controlled life and death pathways. Although the ER-positive BCs expressed ample IAPs, even more than the TN-BCs, only the latter were killed by SMs. The authors explored additional factors related to the IAPs and cytokine death-ligand pathways by measuring mRNA levels in the TN-BC compared with the ER-positive patient-derived xenografts. Furthermore, they analyzed the publicly available RNA expression data for TN-BC versus ER-positive BC in The Cancer Genome Atlas (TCGA) as well as METABRIC databases. Interestingly, there were several notable differences in mRNA levels for critical factors that may help explain the differences between TN-BC and ER-positive BC for the SM-mediated sensitization of TN-BC to death ligands from the immune system. Notable amongst these differences were that the TN-BCs expressed more TNF-α and its receptor, TNFR1, than ER-positive BCs, and that the TN-BCs expressed less of the death-inducing components of the TNF/TNFR1 pathway, specifically caspase-3 and -8, FADD, RIPK1, and RIPK3, compared with ER-positive BC. This would suggest that TN-BCs have a higher reliance upon the TNF/TNFR1/TRADD/RIPK1/TRAF2/cIAP1-2/LUBAC/IKK/NF-κB survival axis to promote growth and avoid TNF-α mediated apoptosis or necroptosis outcomes, compared with ER-positive BCs (Fig. 1). However, SMs can undermine this TNF-α dependency of TN-BCs and promote TNF-induced killing of those cancers even though there is a relative reduction in the death effector levels. Additional findings from the mRNA analyses that support the cell line observations of death-ligand sensitivity indicate that the TRAIL death receptors, DR4 and DR5, as well as the FasL death receptor, Fas/ CD95, are upregulated in TN-BCs. These other RIPK1/FADD/caspase-8 death pathways do not depend on cIAP1/ 2 (unlike TNFR1), they are inhibited by XIAP at the very distal end of caspase-3 and -7 activation and this too can be overcome by SM antagonism of XIAP function (Fig. 1). One apparently paradoxical observation is the upregulation of the MLKL pore-forming protein and effector of necroptotic cell death observed in TN-BCs. However, this is matched in TN-BCs by a downregulation of RIPK3, the kinase needed to phosphorylate the inactive MLKL and trigger its oligomerization and death-inducing properties by disruption of the plasma membrane (Fig. 1). Dysregulation of RIPK3 and MLKL levels, and inactivation of this inflammatory cell-death pathway, is commonly seen in numerous cancers (e.g.^7–9^). For example, the induction of MLKL may be caused by the immune infiltrate and IFN production^8,9^. While RIPK1, which acts in concert with RIPK3 to form fibrils and phosphorylate MLKL, is consistently preserved in cancers because it is also required for the operation of the TNF/TNFR1/NF-κB signaling axis. This duality of RIPK1 function, life or death fates depending on RIPK1’s ubiquitination status mediated by the cIAPs, is what allows SMs to toggle so efficiently between these TNFα-mediated outcomes on cancer cells (Fig. 1). This is also coupled with the maintenance of caspase-8 expression that has both prodeath and prosurvival functions, as caspase-8 cleaves RIPK1 and RIPK3 to suppress necroptosis^10^ and has other mitotic roles as well^11^. Although not directly investigated in the Lalaoui report, another possible reason for TRAF2 upregulation and cIAP1/2 involvement in NF-κB activation in TN-BC is their additional involvement in the oncogenic IKKε pathway^12,13^.

## TN-BC profiling and translation of targeted therapies into the clinic

In a final series of experiments^6^, the authors combine the SM birinapant with the taxane, docetaxel which is used for BC therapy and which is also known to induce TNF-α, in their TN-BC models. The combination demonstrates synergy in TN-BC killing in vitro and produces TN-BC tumor regression and long-term survival of mice when either single agent fails to do so. The previous gene expression findings, as well as the positive effects of the combination, are in line with a recent clinic trial combination of the monomeric SM, LCL161, with the taxane paclitaxel in TN-BC patients^14^. This trial showed an improvement in pathologic complete response for the SM combination compared with paclitaxel alone. Importantly, the observed increased effects of the combination therapy related to patients’ expression, a gene signature that primarily relied upon high TNF-α and high RIPK1 in the responding TN-BCs^14^.

The use of targeted cancer agents such as tyrosine kinase inhibitors against oncogenic receptor tyrosine kinases, or immunotherapies such as the anti-PD1 biologics, typically rely on the determination of the presence of the drug target, as assessed by immunohistochemistry, mRNA expression, or DNA sequencing, as a prerequisite for treating cancer patients with those targeted therapies. Hence, the determination of the TNF-α/RIPK1 pathway expression levels in tumors could support the use of SM therapies for specific TN-BC cohorts. This will complement other such targeted therapies for TN-BC, such as PARP inhibitors for BRCA1/2 mutations and immune checkpoint inhibitors for other cohorts of TN-BC expressing PD-L1^3,15^. These advances using therapeutics against novel TN-BC pathways will produce improved outcomes for select cohorts of TN-BC that can be included as BCs for which targeted therapy is a new option.
